# Effect of biological variation in HbA1c and blood glucose on the diagnosis of prediabetes

**DOI:** 10.1002/edm2.442

**Published:** 2023-07-26

**Authors:** James M. Hempe, Shengping Yang, Daniel S. Hsia

**Affiliations:** ^1^ Department of Pediatrics Louisiana State University Health Sciences Center New Orleans Louisiana USA; ^2^ Pennington Biomedical Research Center Baton Rouge Louisiana USA

**Keywords:** blood glucose, glycated haemoglobin, haemoglobin glycation index, prediabetes

## Abstract

**Introduction:**

People with a low or high haemoglobin glycation index (HGI) have lower or higher HbA1c than other people with the same FPG. This study compared the prevalence of prediabetes based on FPG, 2hOGTT and HbA1c in people with low, moderate or high HGI.

**Methods:**

Prediabetes was diagnosed based on ADA cutpoints in 10,488 NHANES participants without self‐reported diabetes. HGI was calculated as the difference between a participant's observed HbA1c and a predicted HbA1c where predicted HbA1c = 0.024 FPG + 3.1. Participants were divided into low (HGI < −0.15%), moderate (HGI −0.15% to +0.15%) and high (HGI > +0.15%) HGI subgroups.

**Results:**

The prevalence of prediabetes was 42.4% based on FPG, 27.2% based on HbA1c and 17.2% based on 2hOGTT. FPG and HbA1c thus overdiagnosed prediabetes by 25.2% and 10.0%, respectively, compared to the OGTT gold standard. Prevalence was (1) similar in low, moderate and high HGI participants based on 2hOGTT, (2) highest in low HGI participants based on FPG, and (3) highest in high HGI participants based on HbA1c. Among participants with mismatched FPG and HbA1c, OGTT was normal in (1) 79.5% of participants with normal FPG but prediabetic HbA1c (mean HGI = +0.53%), and (2) 75.2% of participants with normal HbA1c but prediabetic FPG (mean HGI = −0.30%).

**Conclusions:**

FPG overdiagnosed prediabetes in people with low HGI. HbA1c overdiagnosed prediabetes in people with high HGI. Clinical use of HGI could improve prediabetes diagnosis and help health care providers avoid inappropriate or delayed treatment of people with extremes of HGI.

## Graphical abstract

The prevalence of prediabetes based on 2hOGTT was similar in NHANES participants with low, moderate or high HGI. The prevalence of prediabetes based on FPG was disproportionally higher in low HGI participants while the prevalence of prediabetes based on HbA1c was disproportionally higher in high HGI participants. HGI innovatively incorporates HbA1c and FPG into one value that simplifies risk assessment and limits over diagnosis or underdiagnosis of prediabetes.

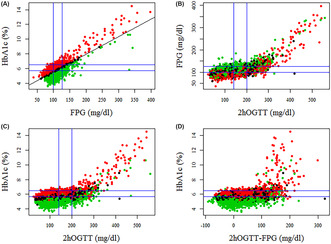

**Abbreviations**
2hOGTT: oral glucose tolerance test
FPG: fasting plasma glucose
HbA1c: glycated haemoglobin A1c
HGI: haemoglobin glycation index

## 1 INTRODUCTION

### 1.1 The diagnostic dilemma

Diabetes can be clinically diagnosed based on fasting plasma glucose (FPG), 2‐hour plasma glucose following a 75 g oral glucose tolerance test (2hOGTT) or glycated haemoglobin A1c (HbA1c). All three metrics are continuous variables in human populations with no inflection points that differentiate between normal glucose metabolism and chronic hyperglycaemia. Marked overlap in what's normal and what's abnormal led to the use of ‘prediabetes’ as a clinical diagnosis for individuals with inconclusive test results. Normal, prediabetic and diabetic reference ranges recommended by the American Diabetes Association (ADA) represent the medical community's best estimate of what can be expected in metabolically normal people compared to those with Type 2 diabetes.^1^ Periodic prediabetes screening is now recommended for all people between 35 and 70 years of age based on FPG, 2hOGTT or HbA1c.^2^ The problem with this multimetric approach is that prediabetes diagnoses based on these three common clinical tests often disagree.^3^, ^4^, ^5^, ^6^

### 1.2 Are two metrics better than one?

In an effort to develop a more specific, timely and unambiguous approach to diabetes diagnosis, Selvin et al.^7^ proposed a two‐dimensional, single‐sample definition of *confirmed undiagnosed diabetes* as having both FPG and HbA1c levels above their respective ADA diabetes cutpoints. The investigators used this confirmation‐by‐agreement approach to analyse data from the Atherosclerosis Risk in Communities study and found that ‘A single‐sample confirmatory definition of diabetes had a high positive predictive value for subsequent diagnosis and was strongly associated with clinical end points’. The authors defined *unconfirmed undiagnosed diabetes* as having either FPG or HbA1c but not both above their respective cutpoints and suggested that diagnostic ambiguity should be addressed by monitoring FPG and HbA1c over time.

### 1.3 Person‐to‐person variation in HbA1c and blood glucose

**FIGURE 1:**
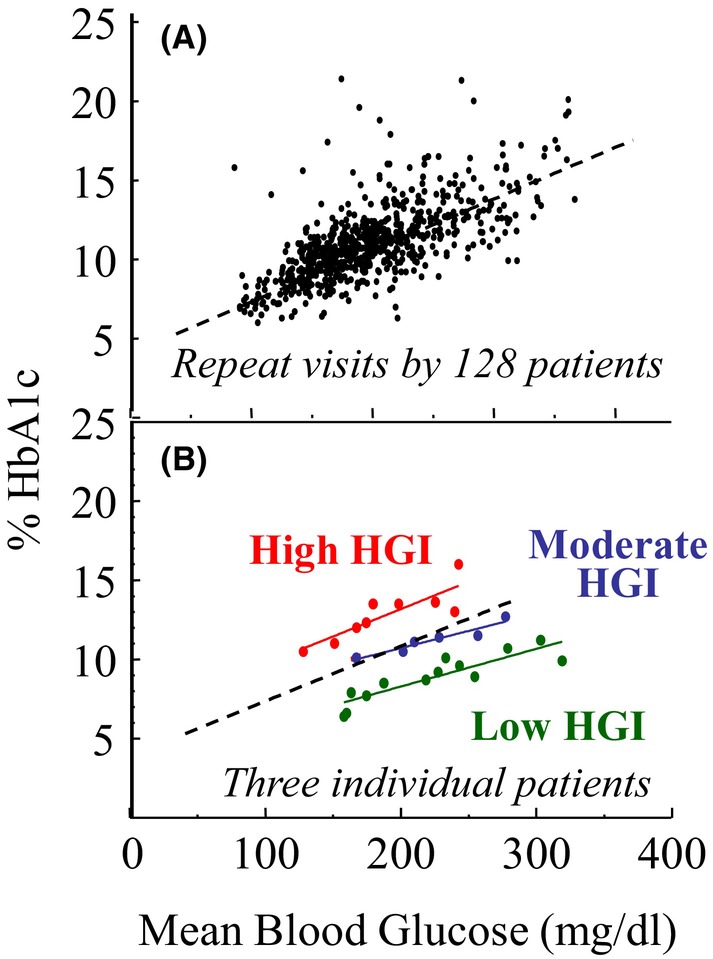
Distribution of paired blood glucose and HbA1c measurements in (A) a longitudinal study of 128 paediatric Type 1 diabetes patients, and (B) repeat observations over time from three of the patients with characteristically low (green), moderate (blue) or high (red) HGI phenotypes.

Figure 1A graphically depicts the linear relationship between blood glucose and HbA1c observed in a 2002 longitudinal study of paediatric of Type 1 diabetes patients.^3^ Figure 1B shows results from three individuals within the population who had HbA1c levels that were consistently lower than average (green), average (blue) or higher than average (red) across a wide range of blood glucose concentrations. Many individuals in the study population had individual regression lines with 95% confidence intervals that did not overlap the population regression line or the confidence intervals of other individuals. A vertical line drawn across all three individual regression lines at any blood glucose level shows that the HbA1c range was about 5%. Similarly, a horizontal line drawn across all three personal regression lines at any HbA1c level shows that the blood glucose range was about 200 mg/dL. One interpretation is that some people have consistently lower or higher HbA1c levels compared to other people with the same blood glucose concentration. Alternatively stated, some people have consistently lower or higher blood glucose concentrations compared to other people with the same HbA1c. Either way, persistent person‐to‐person variation in the quantitative relationship between blood glucose and HbA1c is a fact of nature that complicates the clinical diagnosis of prediabetes and diabetes.

### 1.4 The Haemoglobin Glycation Index (HGI) measures person‐to‐person variation in HbA1c and blood glucose

HGI was introduced as a way to quantify the magnitude and direction of population variation in the quantitative relationship between blood glucose and HbA1c.^3^ HGI is calculated as the difference between an individual's observed HbA1c and a predicted HbA1c obtained by inserting a date‐matched blood glucose concentration into a linear regression equation describing the quantitative relationship between HbA1c and blood glucose in a reference population. This approach naturally incorporates information contained in HbA1c and FPG measurements into a single value. As reviewed in greater detail elsewhere^8^, ^9^ HGI varies between races, ranges from about −2.0% to +2.0% in populations without known diabetes and tends to be consistent within individuals over time and over the physiological range of blood glucose concentrations (Figure 1B). HGI is simple to calculate and has potential clinical application because high HGI is associated with both (1) greater risk for hypoglycaemia in people with diabetes, and (2) greater risk for chronic vascular disease in people with or without diabetes.

## 2 METHODS

This study compared single‐metric and two‐dimensional diagnosis of prediabetes and diabetes based on ADA cutpoints for FPG, 2hOGTT and HbA1c in people with low, moderate or high HGI.

### 2.1 Study population

Data from the 1999–2016 cohorts of the National Health and Nutrition Examination Survey (NHANES) were used to identify participants who (1) were at least 20 years of age, (2) had FPG, 2hOGTT and HbA1c data and (3) had not been previously diagnosed with diabetes. NHANES is an ongoing national survey directed by the Centers for Disease Control that uses a stratified multi‐stage probability sampling design to represent the non‐institutionalized U.S. civilian population.^10^ The National Centers for Health Statistics Ethics Review Board approved the NHANES study protocol and each participant provided written informed consent.

### 2.2 Calculating HGI

HGI is the mathematical difference between an individual's observed HbA1c and a predicted HbA1c calculated by inserting FPG into a linear regression equation (predicted HbA1c = 0.024 FPG + 3.1) derived from a reference population of 18,675 adult participants in the 1999–2016 NHANES cohorts who had not been previously diagnosed with diabetes.^9^ The current study population was subdivided into three roughly equal sized groups of participants with low (HGI < −0.15%), moderate (HGI −0.15% to +0.15%) or high (HGI > +0.15%) HGI based on cutpoints derived from the reference population.

### 2.3 Diagnosis of prediabetes and diabetes

Single‐metric diagnosis of prediabetes and diabetes was based on the ADA cutpoints listed in Table 1. The two‐dimensional confirmation‐by‐agreement approach used by Selvin et al.^7^ for the diagnosis of confirmed undiagnosed diabetes was extended to include the diagnosis of *confirmed normal* and *confirmed undiagnosed prediabetes* using two‐dimensional combinations of FPG, 2hOGTT and HbA1c. The use of three diagnostic outcomes (normal, prediabetic and diabetic) for each pair of diagnostic metrics (FPG + HbA1c, FPG + 2hOGTT and 2hOGTT + HbA1c) produced three nine‐compartment models (Table 2A–C) that include (1) three diagonal compartments from bottom left to top right with confirmed normal, confirmed prediabetes or confirmed diabetes diagnoses, and (2) six compartments (three above and three below the confirmed box in each column) with ambiguous or ‘mismatched’ diagnoses where the two metrics disagree.

**TABLE 1:** Single‐metric diagnosis of prediabetes and diabetes in the NHANES study population based on the ADA cutpoints for FPG, HbA1c and 2hOGTT.

HGI	*N*	Diagnosis (%)		
		Normal	Prediabetes	Diabetes
FPG mg/dL		<100	100–125	≥126
All	10,488	**53.3**	**42.4**	**4.3**
Low	3487	43.2	51.0	5.8
Moderate	3507	55.5	42.0	2.5
High	3494	61.1	34.4	4.5
HbA1c %		<5.7	5.7–6.4	≥6.5
All	10,488	**70.2**	**27.2**	**2.6**
Low	3487	94.6	4.8	0.6
Moderate	3507	81.8	17.3	0.9
High	3494	34.1	59.6	6.3
2hOGTT mg/dL		<140	140–199	≥200
All	10,488	**76.3**	**17.2**	**6.5**
Low	3487	79.0	16.2	5.5
Moderate	3507	79.0	16.1	4.9
High	3494	70.8	19.9	9.2

*Note:* Bold values are focused on in the text.

**TABLE 2:** Two‐dimensional diagnosis of prediabetes and diabetes in the NHANES study population based on the nine compartments formed by the combined ADA cutpoints for (A) FPG + HbA1c, (B) FPG + 2hOGTT and (C) HbA1c + 2hOGTT.

**A**	**Normal FPG < 100 mg/dL**	**Prediabetes FPG 100–125 mg/dL**	**Diabetes FPG ≥ 126 mg/dL**
Diabetes HbA1c ≥ 6.5%	*n* = 3 <0.0% HGI = +1.37	*n* = 85 0.8% HGI = +0.83	*n* = 185 **1.8%** **HGI = +0.63**
Prediabetes HbA1c 5.7 to 6.4%	*n* = 940 9.0% HGI = +0.53	*n* = 1734 **16.5%** **HGI = +0.24**	*n* = 183 1.7% HGI = −0.21
Normal HbA1c < 5.7%	*n* = 4644 **44.3%** **HGI = −0.05**	*n* = 2632 25.1% HGI = −0.30	*n* = 82 0.8% HGI = −0.90
**B**	**Normal FPG < 100 mg/dL**	**Prediabetes FPG 100–125 mg/dL**	**Diabetes FPG ≥ 126 mg/dL**
Diabetes 2hOGTT ≥ 200 mg/dL	*n* = 53 0.5% HGI = +0.23	*n* = 344 3.3% HGI = +0.10	*n* = 287 **2.7%** **HGI = +0.25**
Prediabetes 2hOGTT 140–1 99 mg/dL	*n* = 559 5.4% HGI = +0.15	*n* = 1146 **10.9%** **HGI = 0.0**	*n* = 99 0.9% HGI = −0.32
Normal 2hOGTT < 140 mg/dL	*n* = 4976 **47.5%** **HGI = +0.04**	*n* = 2960 28.2% HGI = −0.11	*n* = 64 0.6% HGI = −0.53
**C**	**Normal 2hOGTT < 140 mg/dL**	**Prediabetes 2hOGTT 140–199 mg/dL**	**Diabetes 2hOGTT ≥ 200 mg/dL**
Diabetes HbA1c ≥ 6.5%	*n* = 26 0.2% HGI = +0.86	*n* = 41 0.4% HGI = +0.68	*n* = 206 **2.0%** **HGI = +0.69**
Prediabetes HbA1c 5.7%–6.4%	*n* = 1739 16.6% HGI = +0.36	*n* = 796 **7.6%** **HGI = +0.29**	*n* = 322 3.1% HGI = +0.10
Normal HbA1c < 5.7%	*n* = 6235 **59.4%** **HGI = −0.13**	*n* = 967 9.2% HGI = −0.21	*n* = 156 1.5% HGI = −0.37

*Note*: The number of observations, proportion of the population and the participants mean HGI are listed for each compartment.

Bold values are focused on in the text.

### 2.4 Data analysis

Descriptive statistics were used to describe demographic and other characteristics of the study cohort. Categorical variables were summarized as frequencies, and continuous variables were summarized as appropriate using means and standard deviations or ranges. Data stratification was performed using the recommended ADA cutpoints. Data distributions were presented in histograms and scatter plots. Analyses were performed using SAS (Windows version 9.4; SAS Institute) and the statistical program R version 4.0.2 (Version 3.3.2; R Core Team).

## 3 RESULTS

### 3.1 Distribution of FPG, 2hOGTT, HbA1c and HGI in the NHANES study population

**FIGURE 2:**
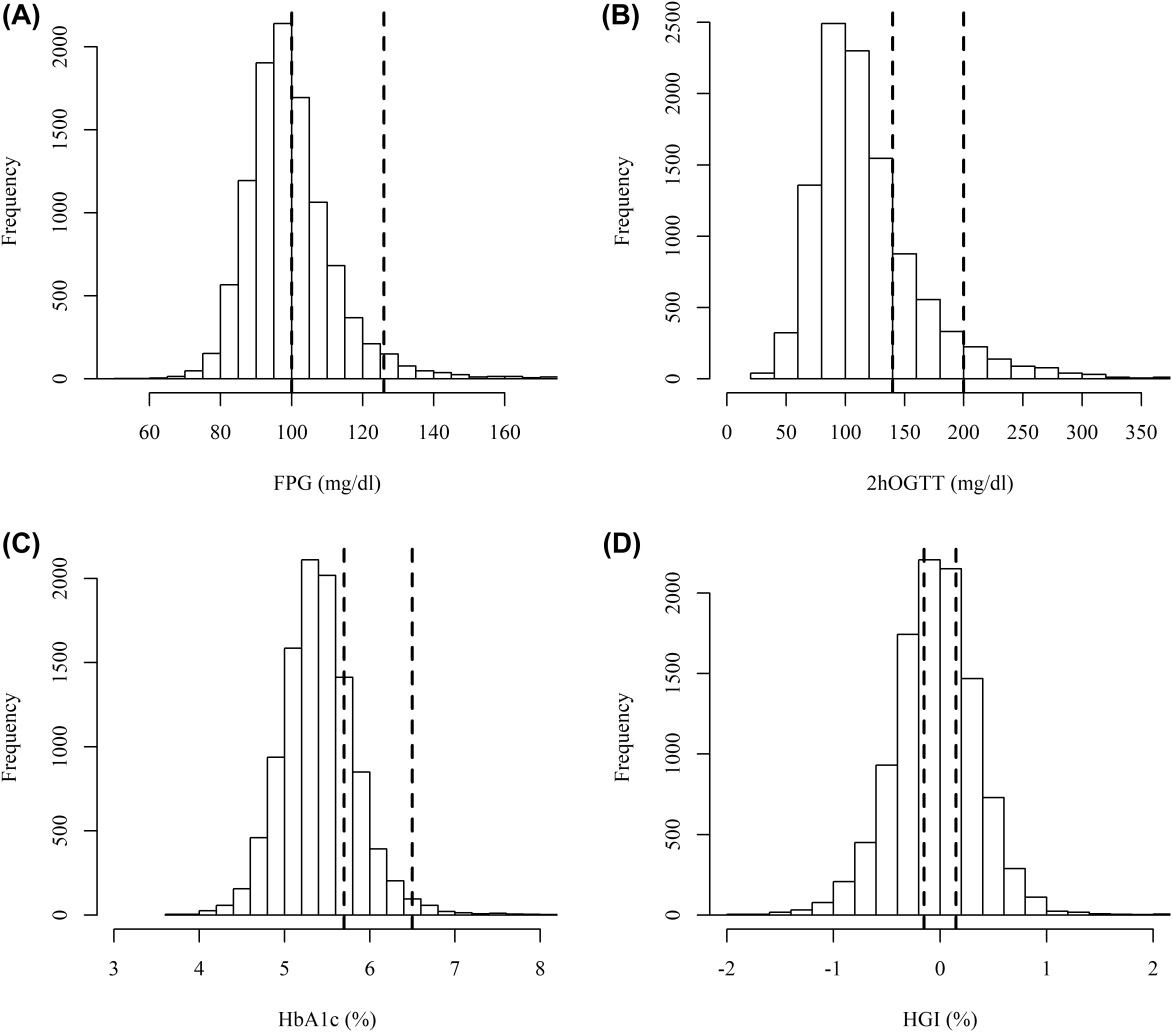
Distribution of FPG (A), 2hOGTT (B), HbA1c (C) and HGI (D) in NHANES participants without self‐reported diabetes. Dotted lines highlight the locations of the prediabetes and diabetes cutpoints and the HGI cutpoints that divide the study population into equal sized low, moderate and high HGI subgroups.

Figure 2 shows that FPG, 2hOGTT, HbA1c and HGI were continuous quantitative variables with wide physiological ranges, normal or slightly right‐skewed distribution curves in NHANES participants without self‐reported diabetes.

### 3.2 Single‐metric diagnosis of prediabetes and diabetes

Table 1 shows that (1) FPG diagnosed 53.3% of the 10,488 NHANES participants as normal, 42.4% as prediabetic and 4.3% as diabetic, (2) HbA1c diagnosed 70.2% as normal, 27.2% as prediabetic and 2.6% as diabetic and (3) 2hOGTT diagnosed 76.3% as normal, 17.2% as prediabetic and 6.5% as diabetic. The prevalence of prediabetes in low, moderate and high HGI participants based on FPG, 2hOGTT and HbA1c varied significantly, where (1) FPG diagnosed 51.0% of low, 42.0% of moderate and 34.4% of high HGI participants as prediabetic, (2) HbA1c diagnosed 4.8% of low, 17.3% of moderate and 59.6% of high HGI participants as prediabetic and (3) 2hOGTT diagnosed 16.2% of low, 16.1% of moderate and 19.9% of high HGI participants as prediabetic.

### 3.3 Graphical evaluation of diagnostic cutpoints

**FIGURE 3:**
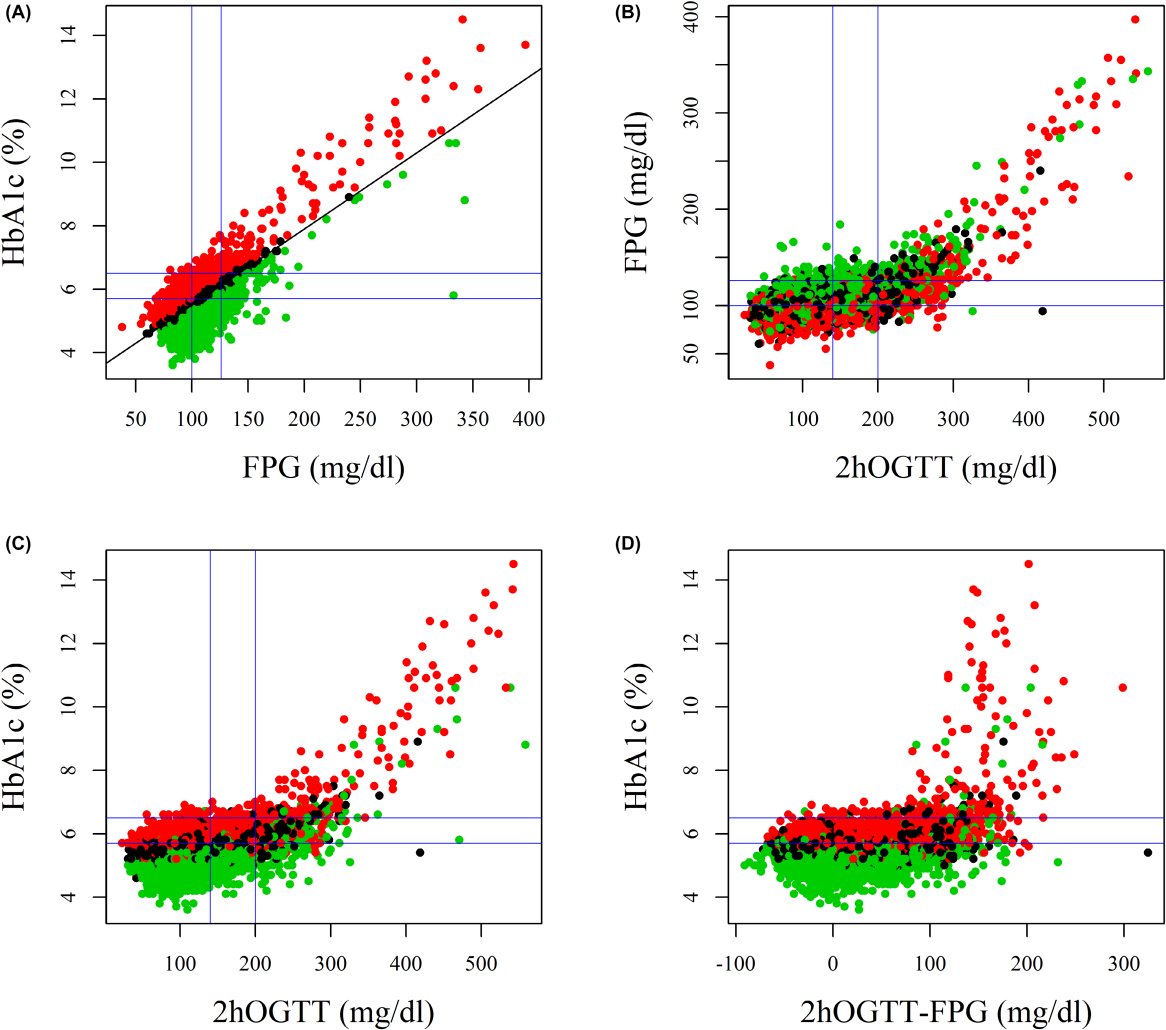
Scatterplots and linear regression lines for paired FPG, HbA1c and 2hOGTT observations in NHANES participants in the low (green), moderate (blue) or high (red) HGI subgroups overlaid with intersecting blue lines depicting the two‐dimensional nine‐compartment models defined by each metric's ADA prediabetes and diabetes cutpoints.

Figure 3A–C are two‐dimensional graphs of paired combinations of FPG, 2hOGTT and HbA1c in the 10,488 NHANES study participants overlaid with blue lines representing each metric's ADA cutpoints for prediabetes and diabetes. Figure 3D graphs the relationship between HbA1c and postchallenge glucose increment (2hOGTT‐FPG).

### 3.4 Two‐dimensional diagnosis of prediabetes and diabetes

Table 2 summarizes the number of observations and mean HGI of the three nine‐compartment diagnostic models produced by the two‐dimensional combinations of FPG, 2hOGTT and HbA1c as depicted in Figure 3A–C. The combination of FPG + HbA1c (Table 2A) produced 62.6% confirmed diagnoses (44.3% normal, 16.5% prediabetes, 1.8% diabetes) and 37.4% ambiguous or mismatched diagnoses. The combination of FPG + 2hOGTT (Table 2B) produced 61.1% confirmed diagnoses (47.5% normal, 10.9% prediabetes, 2.7% diabetes) and 38.9% ambiguous diagnoses. The combination of 2hOGTT + HbA1c (Table 2C) produced 69.0% confirmed diagnoses (59.4% normal, 7.6% prediabetes, 2.0% diabetes) and 31.0% ambiguous diagnoses. In all three two‐dimensional comparisons, mean HGI was (1) positive in participants with confirmed diabetes, (2) positive in the compartments that lie above the line of diagnostic agreement and (3) negative in the diagnostic compartments below the line.

More granular analysis of the FPG + HbA1c data showed that 47.7% of the study population had 2hOGTT diagnoses that agreed with two‐dimensional confirmed normal, prediabetes and diabetes diagnoses. FPG and HbA1c diagnoses both agreed with 2hOGTT in (1) 91.8% of the 4644 participants diagnosed as confirmed normal (Table 2A), (2) 32.5% of the 1734 participants diagnosed as confirmed prediabetic and (3) 93.5% of the 185 participants diagnosed as confirmed diabetic. Among participants with mismatched FPG and HbA1c diagnoses, 2hOGTT was normal in (1) 79.5% of the 940 participants with normal FPG but prediabetic HbA1c, and (2) 75.2% of the 2632 participants with normal HbA1c but prediabetic FPG.

## 4 DISCUSSION

### 4.1 Biological variation complicates single‐metric diagnosis of prediabetes

Single‐metric diagnosis showed that the ADA cutpoints for FPG and HbA1c overestimate the number of NHANES participants with abnormal glucose metabolism compared to 2hOGTT. Diagnosis based on FPG (Table 1) identified (1) far fewer participants as normal (53.3%) compared to HbA1c (70.2%) or 2hOGTT (76.3%), and (2) far more participants as prediabetic (42.4%) compared to HbA1c (27.2%) or 2hOGTT (17.2%). By subtracting the proportion of NHANES participants diagnosed as prediabetic based on the 2hOGTT gold standard from that diagnosed as prediabetic based on FPG and HbA1c, we conclude that FPG and HbA1c overestimated the prevalence of prediabetes in the study population by 25.2% and 10.0%, respectively.

The range in prediabetes prevalence observed in the low, moderate and high HGI subgroups was 16.6% (34.4%–51.0%) based on FPG, 54.8% (4.8%–59.6%) based on HbA1c, but only 3.8% (16.1%–19.9%) based on 2hOGTT. We conclude that the prevalence of prediabetes based on 2hOGTT was similar in low, moderate and high HGI people as previously reported.^6^, ^11^, ^12^ By subtracting the proportion of low or high HGI participants diagnosed as prediabetic based on 2hOGTT from the proportion diagnosed as prediabetic based on FPG or HbA1c, we further conclude that (1) FPG overestimated the prevalence of prediabetes by 34.8% (51.0% vs. 16.2%) in low HGI participants, while (2) HbA1c overestimated the prevalence of prediabetes by 39.7% (59.6% vs. 19.9%) in high HGI participants.

### 4.2 Biological variation complicates two‐dimensional diagnosis of prediabetes

Table 2 shows that mean HGI was higher in patients with confirmed diabetes in all three two‐dimensional combinations of FPG, 2hOGTT and HbA1c. The combination of 2hOGTT + HbA1c produced (1) the most two‐dimensional confirmed normal diagnoses (59.4%) compared to FPG + 2hOGTT (47.5%) or FPG + HbA1c (44.3%), and (2) the fewest confirmed prediabetes diagnoses (7.6%) compared to FPG + 2hOGTT (10.9%) or FPG + HbA1c (16.5%). All three two‐dimensional combinations produced over 30% ambiguous diagnoses. FPG was a more accurate predictor of prediabetes in high HGI participants based on the observation that 2hOGTT was normal in 79.5% of the 940 mismatched participants diagnosed as normal by FPG but prediabetic by HbA1c (mean HGI = +0.53%). In contrast, HbA1c was a more accurate predictor of prediabetes in low HGI participants based on the observation that 2hOGTT was normal in 75.2% of the 2632 participants diagnosed as normal by HbA1c but prediabetic by FPG (mean HGI = −0.30%).

Diagnoses based on the clinically feasible two‐dimensional combination of FPG + HbA1c (Table 2A) agreed with diagnoses based on 2hOGTT in almost all participants diagnosed as confirmed normal (91.8%) or confirmed diabetes (93.5%). In contrast, only 32.5% of the 1734 participants diagnosed as confirmed prediabetes were diagnosed with prediabetes based on 2hOGTT. Low agreement between FPG and HbA1c diagnoses in participants with confirmed prediabetes is attributable to fact that the area of the central two‐dimensional FPG + HbA1c confirmed prediabetes compartment lies disproportionately above the population regression line (Figure 3A). This upward shift in the alignment of the prediabetes compartment relative to the distribution of paired observations is why the three ambiguous compartments above the diagonal line of confirmed diagnoses included disproportionately fewer participants (*n* = 940) with a positive mean HGI (Table 2A) compared to the three ambiguous compartments below the line which included nearly three times as many (*n* = 2632) participants with a negative mean HGI.

Figure 3B,C shows that the FPG and HbA1c responses to increasing 2hOGTT were both biphasic and characterized by (1) a very slow increase in FPG and HbA1c with increasing 2hOGTT below 200 mg/dL, (2) a rapid increase in FPG and HbA1c above 300 mg/dL 2hOGTT and (3) significant overlap in the two phases between 200 and 300 mg/dL 2hOGTT. Almost all NHANES participants with 2hOGTT levels below 200 mg/dL, which includes both normal and prediabetes reference ranges, had HbA1c <7.0% (Figure 3C). Figure 3D shows there was little or no quantitative relationship between HbA1c and postchallenge glucose increment in the HbA1c response to increasing 2hOGTT‐FPG. We conclude that there is a wide range in postchallenge glucose increment over which the magnitude of glycaemic excursion had little or no effect on HbA1c in participants with low, moderate or high HGI.

### 4.3 Clinical use of HGI

Staimez et al.^13^ recently reported a study of NHANES participants from the HGI perspective and concluded that ‘Complementing HbA1c measurement with glucose testing may enhance diabetes classification across individuals and reduce the risk of over‐ or undertreatment for diabetes in the United States’. The investigators reached this conclusion using a complex set of nine regression equations, one for each of nine different subpopulations created by dividing participants into three age groups (youth, adults and older adults) with three diagnoses (normal, prediabetes or diabetes). Also, instead of FPG, the authors used the mean of FPG and 2hOGTT to generate the nine regression equations and predicted HbA1c. Their results confirmed previously reported demographic and clinical observations, including higher HGI in black, obese and older people and no difference in 2hOGTT between low, moderate and high HGI subgroups.

To investigate how HGI might be used clinically, Staimez et al.^13^ classified individuals as having a significant mismatch between HbA1c and blood glucose if the absolute value of HGI was 0.5%, that is, individuals with 0.5% higher or lower HbA1c than predicted by FPG. The investigators reported that 10% of youth, 15% of adults and 20% of older adults had clinically significant mismatches. We applied the same HGI mismatch cutpoints to the adult NHANES participants in the present study and found that 7.1% had HGI < −0.5% (mean HGI = −0.73%) and 11.2% of the population had HGI > +0.5% (mean HGI = +0.74%). We conclude that a total of 18.3% of the study population had significant mismatches in FPG and HbA1c and greater risk for misdiagnosis. We further note that calculating HGI based on multiple regression equations and the mean of FPG + 2hOGTT is impractical for clinical use and unnecessary given the similarity of the demographic and clinical results observed by Staimez et al.^13^ to what has previously been reported using a single FPG and a single‐HGI regression equation.

## 5 CONCLUSIONS

Prediabetes was adopted as a clinical diagnosis to account for the fact that FPG, 2hOGTT and HbA1c are all continuous variables with overlapping normal and abnormal physiological ranges. Diagnostic uncertainty creates a Catch‐22 ‘treat or not treat’ predicament for health care providers where a rush to treatment with glucose‐lowering drugs could subject false positives to treatment‐induced hypoglycaemia, while a delay in treatment could subject true positives to the damaging effects of emerging hyperglycaemia. The present study used HGI to explore how the combination of biological variation and fixed diagnostic cutpoints produces uncertainty in the diagnosis of prediabetes based on FPG or HbA1c.

The results show that compared to 2hOGTT, FPG significantly overdiagnoses prediabetes in people with low HGI, while HbA1c overdiagnoses prediabetes in people with high HGI. This research showcases the fact that HGI derived based on two routinely available laboratory tests is a clinically practical single metric that can help take the guesswork out of identifying people with emerging diabetes. More research is needed to determine (1) the degree to which misdiagnosis of prediabetes based on FPG and HbA1c influences the treatment and outcomes of people with extremes of HGI, and (2) how HGI should be used clinically to improve prediabetes diagnosis. We strongly recommend the use of our proposed HGI standardisation equation and cutpoints^9^ as a way to make HGI comparable between hospitals, clinics and research studies.

## AUTHOR CONTRIBUTIONS

**James M. Hempe:** Conceptualization (lead); methodology (equal); project administration (equal); writing – original draft (lead); writing – review and editing (equal). **Shengping Yang:** Data curation (lead); formal analysis (lead); methodology (equal); writing – review and editing (equal). **Daniel S Hsia:** Project administration (equal); writing – review and editing (equal).

## CONFLICT OF INTEREST STATEMENT

The authors have no conflicts to disclose.

## Data Availability

The data that support the findings of this study are openly available through the National Health and Nutrition Examination Survey at https://wwwn.cdc.gov/nchs/nhanes/
